# Botulinum Toxin Treatment for Depression: A New Paradigm for Psychiatry

**DOI:** 10.3390/toxins15050336

**Published:** 2023-05-14

**Authors:** Eric Finzi

**Affiliations:** 1 Healis Therapeutics, 4041 MacArthur Blvd Suite 400, Newport Beach, CA 92660, USA; eric@healisthera.com; 2 Department of Psychiatry, George Washington School of Medicine & Health Sciences, Washington, DC 20037, USA

**Keywords:** botulinum toxin, depression, clinical trials, psychiatry, amygdala, corrugator, emotional proprioception, antidepressant

## Abstract

Multiple randomized double-blind placebo-controlled trials have shown that botulinum toxin A (BoNT/A), when injected into the frown musculature, is an antidepressant. This review outlines the conceptual narrative behind this treatment modality, starting with theory developed by Charles Darwin. We develop the concept of emotional proprioception and discuss how the muscles of facial expression play an important role in relaying valenced information to the brain’s emotional neuroanatomical circuit. We review the role of facial frown musculature as the brain’s barometer and transmitter of negatively valanced emotional information. The direct connections between the corrugator muscles and the amygdala are reviewed, and these provide a neuroanatomical circuit that is a logical target for treatment with BoNT/A. The centrality of amygdala dysfunction in the pathogenesis of many psychiatric disorders, and the evidence that BoNT/A modulates amygdala activity, provides the mechanistic link between BoNT/A and its antidepressant activity. Animal models of BoNT/A’s antidepressant effects confirm the evolutionary conservation of this emotional circuit. The clinical and theoretical implications of this evidence, as it relates to the potential treatment of a broad range of psychiatric disorders by BoNT/A, is discussed. The ease of administration, long duration, and favorable side effect profile of this therapy is reviewed in the context of existing antidepressant treatments.

## 1. Introduction

Since the first open-label trial [1], multiple randomized double-blind placebo-controlled trials have shown that botulinum toxin A (BoNT/A) is an antidepressant when injected into the corrugator and procerus muscles [2,3,4,5,6,7]. In addition, four other trials have reported similar results [8,9,10,11]. These results have surprised many, but a line of thinking derived from the 19th century predicted them. We usually assume that our emotions affect our facial expressions, but the effect of our facial expressions on our emotions is less apparent. However, there is a large body of evidence that suggests our facial expressions provide a corporeal means to encode and deliver emotional information.

In his prescient and groundbreaking book, “The Expression of Emotions in Man and Animals”, Charles Darwin states, “The free expression by outward signs of an emotion intensifies it. On the other hand, the repression, as far as possible, of all outward signs softens our emotions. He who gives way to violent gestures will increase his rage; he who does not control the signs of fear will experience fear in greater degree” [12]. Darwin described the omega sign (Ω) between the eyebrows, so called because it has the shape of the last letter of the Greek alphabet, in subjects suffering from severe melancholy. He related the case of a woman suffering from severe melancholia who imagined that she had lost her viscera, and that her body was empty. When she spontaneously recovered some months later, he noted that “her countenance resumed its natural expression”.

Darwin suggested that facial expressions were innate and universal among humans, and to strengthen his argument he drew parallels with facial expressions in other primates. He emphasized their important role, given their presence in multiple species. In support of his theory of the primacy of facial expressions, he described emotional expressions in cats, dogs, horses, and nonhuman primates. He also described in detail how infants and the blind form the same expressions.

William James, the Harvard psychologist, was a visionary when he elaborated on his facial feedback theory of emotion in his Principles of Psychology [13]. James stated that he was sad because he cried, rather than cry because he was sad. James suggested that our muscles embody our emotions, and that this embodiment is an intrinsic part of the emotion. He theorized that changes in muscle tension reflect the emotion of the moment, regardless of any conscious awareness. He said, “Smooth the brow, brighten the eye… and your heart must be frigid indeed if it does not gradually thaw”! Both Darwin and James hypothesized that the facial expression representations of sadness signaled to the emotional centers of the brain, creating or worsening feelings of distress.

Almost 100 years after Darwin’s writings Paul Ekman demonstrated that facial expressions were innate and universal to our species [14], establishing their fundamental biological role. He showed that remote tribes in New Guinea, which lacked exposure to any media, and did not have a written language, could instinctively recognize the same emotions expressed by a human face as students at a US university. He concluded that the facial expressions of anger, fear, sadness, disgust, surprise, and happiness were understood by all humans. Anything so innate must serve an important purpose and have strong evolutionary value. Thus, Ekman’s work helped stimulate research in the field of facial expressions.

Ekman’s group [15] asked whether the altering of one’s facial expression could affect our autonomic nervous system. To reduce conscious awareness, subjects were given sets of instructions, such as “raise your brows and pull them together”. They measured involuntary changes in the autonomic nervous system, such as heart rate, finger temperature and skin resistance. In their study with 16 subjects, heart rate increased more in anger and fear than in happiness. Left and right finger temperatures increased more in anger than in happiness. Everyone was assessed before and after directed facial action tasks.

The changes in heart rate associated with anger, fear, and sadness were all significantly greater (*p* < 0.05) than those for happiness, surprise, and disgust. For finger temperature, the change with anger was significantly different (*p* < 0.05) from that of all other emotions. Skin resistance was able to distinguish between sadness, fear, anger, and disgust, with the largest decreases in skin resistance occurring in sadness > anger > fear. Disgust increased skin resistance.

They performed a comparison of the effects of facial expression versus conscious thought on the autonomic nervous system. Subjects were asked to relive a past emotional experience, and then they measured the same aspects of the autonomic nervous system. The results showed that moving facial muscles to create a fearful facial expression had a stronger effect on the heart rate, finger temperature, and skin resistance than reliving a past experience that was fearful. The more powerful effect of a facial expression versus a thought was found for all the other fundamental emotions. However, the level of significance was not reported for the difference between a facial action task and reliving an emotional experience.

Subsequently, a variety of research groups investigated how the muscles of facial expression affect one’s emotional states. In one experiment, to test the effect of frowning on the experience of emotion, researchers placed golf tees on both sides of subjects’ foreheads [16]. They then asked them to move the tees closer together, an action that can only be accomplished by frowning. Subjects were then shown photographs with emotional content. When the golf tees were drawn closer together (creating the frown expression), subjects assessed unpleasant photographs more negatively, showing that the act of frowning affects the emotional valence of our decision making.

Different groups of researchers have come to similar conclusions by using different experimental methods to alter facial expressions. One group noted that the pronunciation of the U vowel in German causes contraction of the frown musculature. Reading stories that contained more U words would lead to more involuntarily frowning, and this led subjects to evaluate stories more negatively. Subjects liked stories that contained no Us better than stories with Us. The authors concluded that facial muscle movement alone, without any conscious awareness, is sufficient to affect the valence of our emotional evaluation of a story [17,18].

One of the most accurate and well-established measures to objectively analyze negative emotions is facial electromyography (EMG) of corrugator muscles. Activity in the corrugator muscles reflects valence-specific negative affect [19,20]. When subjects imagined sad or angry situations, distinctive EMG patterns of muscle activity over the corrugator muscles was observed [21]. Corrugator muscle contraction is essential for frowning and has been demonstrated to directly correlate with subjects’ experience of affect. It decreases with positive emotional states and increases with negative emotional states. Therefore, one might expect that depressed subjects might have elevated corrugator muscle activity. This experiment has been done; EMG has shown that corrugator muscle activity is increased in depression [21,22,23]. In addition, the patterns of EMG activity in addition to intensity were able to distinguish those with depression from the nondepressed [21]. Twelve healthy subjects were compared with twelve depressed subjects. Corrugator activity between the two groups was significantly different, *p* < 0.05, after subjects imagined a happy, sad or angry situation.

Researchers then asked whether EMG patterns of facial muscle activity could predict the treatment outcome for patients suffering from depression. Several groups have shown that the EMG patterns of facial muscle activity are an independent predictor of treatment results [24,25,26].

## 2. Emotional Proprioception

How do we explain the role of the corrugator muscle in emotion? How does movement of the face cause the emotional state?

It has been proposed that afferent nerve fibers from facial musculature relay emotional information to the brain on a constant basis, signaling our emotional state, by a process termed emotional proprioception [27,28,29,30]. In this model, the brain utilizes feedback from facial muscles to provide emotional information. When we paralyze frowning with BoNT/A this initiates a signal to the proprioceptive fibers of the optic branch of the trigeminal nerve. This signal in turn is relayed to the mesencephalic nucleus, which connects to the amygdala and the ventromedial prefrontal cortex [31]. Both brain centers are intimately involved in emotional regulation, and in current models of depression. The amygdala in turn has strong reciprocal connections to the hypothalamus and brain stem regions, which are involved in control of autonomic nervous system functions. Thus, there is a neuroanatomical circuit by which facial muscle contraction affects the autonomic nervous system, as posited by Ekman.

The fundamental connection between the human amygdala and the corrugator muscle has been demonstrated in vivo [32]. Direct intracerebral stimulation of the human amygdala increases corrugator muscle activity.

Corrugator EMG activity is also increased in response to the viewing of negatively valenced pictures [20]. At the same time, a decrease in ventromedial prefrontal cortex activity is seen by fMRI [20]. Thus, there is a reciprocal relationship between the ventromedial prefrontal cortex and the amygdala, and their activity is directly tied to the emotion encoded by the muscles of facial expression. 

### 2.1. The Amygdala Is Central for Negative Emotions and Involved in the Pathogenesis of Most Psychiatric Disease

The negative emotions of sadness, anger and fear are all regulated by the amygdala. The last few decades of research have shown that negative emotions are a fundamental part of a variety of psychiatric disorders.

The amygdala has been shown to be central to the pathogenesis of many psychiatric disorders. For example, brain scans reliably show that amygdala activity is dysregulated in major depressive disorder (MDD) [33]. In addition, post-traumatic stress disorder, social anxiety disorder, bipolar disorder, panic disorder, borderline personality disorder, and obsessive-compulsive disorder have all been reported to have amygdala dysfunction [34,35,36,37,38,39]. Treatments that induce rapid antidepressant effects are also reported to quickly downregulate the amygdala.

More than 30 years of research have confirmed the centrality of the amygdala for the genesis of negative emotions. The amygdala neuronal circuit is one of the most studied neuroanatomical circuits in the brain. This circuit is central to models of depression and post-traumatic stress disorder. For example, an fMRI study in MDD patients treated with paroxetine revealed that amygdala activity was decreased only in those MDD patients who responded to paroxetine; non responders did not down-regulate their amygdala [40], thus directly linking improvement of depression symptoms to amygdala regulation.

### 2.2. Downregulation of the Amygdala

The injection of BoNT/A into the corrugator muscles of depressed subjects is a clear and specific test of more than 30 years research on the pathogenesis of depression.

This research has shown that the amygdala–ventromedial prefrontal cortex neuronal pathway is dysfunctional in MDD. BoNT/A injection at sufficient dose into the glabellar region leads to a temporary but complete inactivation of the procerus and corrugator muscles. Subjects who received BoNT/A injections into the corrugator and procerus muscles had modulation of amygdala activity, as demonstrated by fMRI [41,42]. When subjects view photographs of angry faces, amygdala activity is normally increased; this amygdala response was decreased when the procerus and corrugator muscle contraction was inhibited by BoNT/A injection. Researchers then tested the amygdala response after sufficient time had elapsed such that the effect of BoNT/A on muscular contraction had completely worn off. They found that amygdala activity had returned to its original state. These results confirm that BoNT/A, in a reversible manner, can downregulate amygdala activity [42].

**Figure 1:**
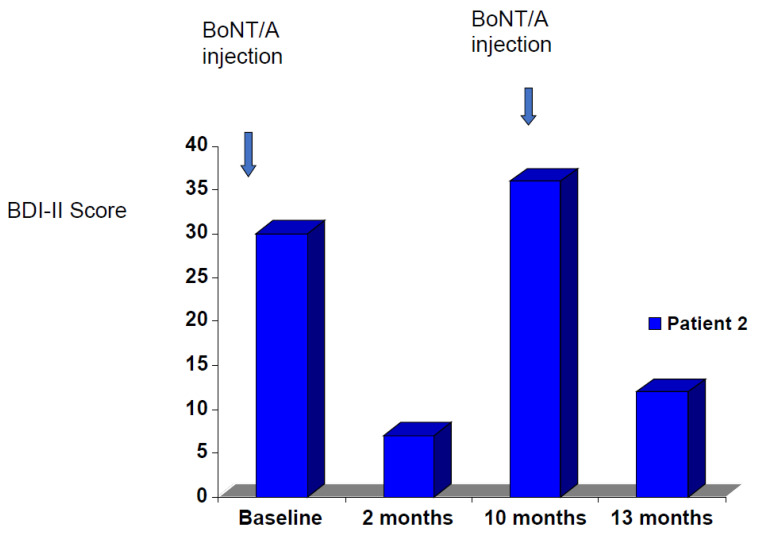
Patient BDI-II scores after BoNT/A treatment.

The reversible nature of BoNT/A modulation of amygdala activity is consistent with the reversible nature of BoNT/A’s clinical results in the treatment of depression. A patient with chronic MDD was treated with BoNT/A; her depression went into remission (Figure 1). She missed her three-month follow-up appointment. When she was seen again at 10 months her depression had returned completely, and her corrugator muscles had returned to their baseline activity. Retreatment with BoNT/A then had a similar antidepressant effect.

Recent research has shown that amygdala activity is also modulated in borderline personality disorder patients treated with BoNT/A [43], suggesting the potential utility of BoNT/A for treatment of borderline personality disorder.

Thus, we now have three independent research groups that have confirmed that BoNT/A injection of the corrugator musculature modulates amygdala activity.

If the concept of emotional proprioception is indeed intrinsic to the mechanism of BoNT/A’s antidepressant effect, then the site of BoNT/A facial injections should be critical for the successful treatment of depression. Thus, BoNT/A injection of frown muscles should improve symptoms of depression, but injection into smile muscles should not. Recently, researchers have performed this experiment. In a single-blind randomized trial of BoNT/A injections for treatment-resistant depression, researchers compared the effectiveness of glabellar versus periorbital injection. Periorbital injection targets the orbicularis oculi muscles, which are part of the smile facial expression. Notably, glabellar, but not perioribital, injection was effective in treating depression [11].

Although existing evidence points towards emotional proprioception as the mechanism of BoNT/A’s antidepressant effect, this does not prove that other mechanisms are not involved. Other possibilities include improved body image and self-esteem due to cosmetic effects. However, this appears less likely since both glabellar and orbicularis injections can cause cosmetic improvements, but only glabellar injections had significant antidepressant effects. In addition, the rodent models of BoNT/A for depression, as described below, make this less likely.

Two major models of emotional transmission, James–Lange and Cannon–Bard, were proposed more than 100 years ago [13,44,45,46,47]. The James–Lange theory of emotion suggests that bodily reactions themselves elicit conscious emotions. By contrast, the Cannon–Bard theory posits that emotional experience occurs independently of physiological arousal. Arguments against the peripheral model (James–Lange) included the slow transmission of neuronal signals from the periphery, such as from the viscera. In the case of neuronal transmission from the corrugator muscles, nerve conduction velocity is not an argument against the James–Lange theory. The corrugator muscles send their signal to the trigeminal nerve, a large cranial nerve with proximity to the amygdala. The role of facial muscles in emotion is most consistent with embodied theories of emotion [48,49].

The precise mechanisms by which BoNT/A glabellar injections lead to modulation of amygdala activity remain to be elucidated.

### 2.3. Animal Models

Darwin inferred the strong evolutionary importance of facial expressions from their occurrence in a wide variety of species. Animal models of depression have been helpful in understanding the disease. Rodent models have demonstrated the antidepressant activity of approved antidepressants.

Human, but not rodent, drug trials can be significantly affected by the placebo response. Psychiatric trials have higher placebo response rates than other types of clinical trial. Recent research has shown that mice demonstrate a defined set of facial expressions when challenged with emotional events [50]. Three different emotional states, pain (tail shock), disgust (quinine), and pleasure (sucrose), were differentiated by facial expressions. Using a machine learning model, they demonstrated that mouse facial expressions exhibited fundamental features of emotions, such as intensity, valence, generalization, persistence, and flexibility. Next, they used optogenetics to activate specific regions of the insular cortex. Projections from the insular cortex to the amygdala have been shown to affect the emotional state induced by the tasting of different solutions. In their optogenetic model, they found that activation of the anterior insular cortex–basolateral amygdala pathway diminished the mouse facial expression of disgust. They then showed that optogenetically induced facial expressions corresponded temporally and in persistence to those triggered by exposure to quinine (disgust).

The authors concluded that facial expressions were consistent indicators of emotional states and that, in mice, the neuronal circuits involved can be inferred by their facial expressions. This raises the question: does BoNT/A show antidepressant activity in rodent models?

In rodents, it has been reported that a lesion in the median forebrain bundle induced by 6-hydroxydopamine (6-OHDA) provides a suitable model to investigate the depressive-like behaviors seen in Parkinson’s disease [51].

The forced swim test is a standard assay for depression in rats, as shown by the effect of standard antidepressant drugs in this model. In the rat 6-OHDA model of Parkinson’s disease, a 1 ng intrastriatal BoNT/A injection significantly reduced depressive behavior, as demonstrated by the forced swim test, *p* < 0.002 [51].

Another rodent model of depression assesses the duration of immobility in space-restricted animals. In mice, a single facial injection of BoNT/A led to a rapid and sustained improvement of depressed behaviors in space-restricted animals, as observed by a shorter duration of immobility [52]. Expression of multiple neurotransmitters and growth factors was affected by BoNT/A. 5-Hydroxytryptamine levels were increased in the hypothalamus and hippocampus. Brain-derived neurotrophic factor was also increased in the amygdala, hippocampus, hypothalamus, and prefrontal cortex. Both 5-hydroxytryptamine and brain-derived neurotrophic factor expression have been reported to be involved in the pathogenesis of depression; their activity is decreased in depressive states.

In a different mouse model, BoNT/A reduced anxiety-like behaviors and inhibited microglia toll-like receptor 2-mediated neuroinflammation [53]. Inflammation is thought to play a significant role in the genesis and maintenance of depressive behavior.

In summary, there are now multiple rodent models of depression that show that a single injection of BoNT/A into rodents has a strong antidepressant effect.

## 3. Randomized Controlled Trials

There is an extensive clinical trials research literature that shows that BoNT/A is a strong antidepressant.

The first open-label trial demonstrated that injection of 29 μ of BoNT/A into the glabellar frown muscles induced remission of depressive symptoms in multiple female patients [1]. Patients received 7 μ into the procerus muscle, 6 μ bilaterally into the medial part of the corrugator muscles, and 5 μ bilaterally into the lateral part of the corrugator muscles. This dosage is almost 50% higher than that commonly used to treat glabellar wrinkles in women, and was chosen to increase the likelihood of complete muscle inhibition. By contrast, treatment for cosmetic reasons often does not aim for complete muscular inhibition. When the paralyzing effects of BoNT/A wore off, the depressive symptoms returned. Reinjection of BoNT/A led to remission.

This research has been followed by multiple controlled trials of BoNT/A for depression. The first randomized double-blind placebo-controlled trial (RCT) found that 60% of BoNT/A subjects responded (a 50% or greater decrease in depression scores), compared to 13% of placebo (*p* = 0.02) at the primary endpoint of 6 weeks [5]. Patients were followed over 16 weeks. The HAM-D score was reduced by 47% in those treated with BoNT/A, and by 9% in the placebo group (*p* = 0.03). Patients in this trial were suffering from moderate unipolar depression that was partly chronic and treatment resistant. Patients were also on various oral antidepressant medications that were maintained during the trial. The effect size was large (Cohen’s d = 1.28).

A second, larger trial (*n* = 74), lasting 6 weeks, reported a BoNT/A response rate that was 52%, vs. 15% for placebo (*p* < 0.001). Of the 74 subjects, 31 were maintained on their preexisting oral antidepressants. The remission rate of 27% was significant (*p* < 0.02) [3]. The magnitude of the pretreatment frown at rest (without forcible contraction) was not correlated with improvement in depression symptoms. These results suggest that cosmetic improvement in wrinkles did not play a role in the mechanism of action.

The third trial had similar results, with a BoNT/A response rate of 45%, vs. 5% for placebo (*p* = 0.007) [4]. This was a cross-over study; after 12 weeks, the placebo group received BoNT/A. Over the follow-up period of 24 weeks, both groups had significant improvements in depressive symptoms after BoNT/A injections.

The next randomized controlled trial was conducted over 6 weeks, with 28 patients suffering from major depression in Iran. At six weeks, the BoNT/A group was significantly improved versus placebo (*p* < 0.004) [5].

A 24-week phase 2 RCT trial with 120 subjects using 30 μ of BoNT/A reported that the BoNT/A group improved significantly, *p*< 0.05, at 3 and 9 weeks and approached significance at 6 weeks, *p* < 0.053 [6]. However, a concurrent 50 μ trial did not achieve statistical significance. Of note, the placebo response rate in this trial was higher than in all other trials. Their trial also excluded subjects who had previously tried or were currently medicated with oral antidepressants; these exclusion criteria, which were different from the other trials, probably contributed to the high placebo response rate, which can make statistical separation of any antidepressant treatment from placebo difficult.

One trial compared the effect of BoNT/A on anxiety and depression in 90 hemifacial spasm patients versus 90 benign essential blepharospasm subjects. Both groups had improvement in anxiety and depression 2 months after BoNT/A (*p* < 0.05) [7].

A trial in 89 depressed subjects with Parkinson’s disease compared the effect of sertraline versus BoNT/A. BoNT/A was noninferior to sertraline at 2 months and had a lower incidence of adverse reactions (BoNT/A 11% and sertraline 29%, *p* < 0.05) [8].

Another trial compared sertraline with BoNT/A for depression in 76 patients over 12 weeks. Overall, both groups improved similarly; however, the BoNT/A group improved faster and had fewer side effects (15% vs. 33% for sertraline) [9].

A 12-week multicenter trial of 88 patients compared BoNT/A (*n* = 61) to placebo (*n* = 21). BoNT/A demonstrated a significant improvement relative to placebo, *p* < 0.0027 [10].

In a single-blind randomized trial, treatment-resistant depressed subjects, (*n* = 58) were treated with either glabellar frown injections or orbicularis oculi (periorbital) injections. At 6 weeks, depression scores in the BoNT/A group were improved (*p* < 0.004) [11].

In summary, there have been 10 trials in five countries: Germany, USA, China, Iran, and France [2,3,4,5,6,7,8,9,10,11]. No trial designs to date have included sham injections. In 9 of the 10 trials the primary outcome measure was met. All of the trial subjects were treated only once with BoNT/A.

## 4. Meta-Analyses

Another line of evidence consists of recent meta-analyses of clinical trials to date [54,55,56,57,58]. The effect size, or Cohen’s D, of BoNT/A relative to placebo ranged from 0.82 to 1.09 across these five meta-analyses. These results confirm the strong antidepressant effect of BoNT/A. In addition, a meta-analysis of trials of patients with comorbid migraine and depression treated with BoNT/A showed a strong antidepressant effect [58].

## 5. FAERS

Indirect evidence for the antidepressant effect of botulinum toxin can be seen by inverse frequency analysis of reports from the FDA Adverse Effect Reporting System (FAERS). Normally, this reporting system is used to assess the risk of side effects from a drug. However, an inverse analysis can be used to see if a drug reduces the incidence of a condition. An analysis of over 40,000 BoNT/A treatment reports, out of 13 million pos-marketing safety reports, revealed that those patients who received BoNT/A injections for a variety of conditions had a significantly lower number of depression reports compared to patients undergoing different treatments for their conditions [59,60]. It is notable that the only other drug so strongly inversely correlated with reporting of depression was ketamine, whose isomer, esketamine, was recently approved for treatment-resistant depression.

More recently, post-marketing safety surveillance data was investigated to determine whether users of BoNT/A had a reduced incidence of anxiety. Users of BoNT/A reported a lower incidence of new anxiety than did nonusers. Thus BoNT/A appears to have some protective effect against anxiety [61]. However, direct trials will be necessary in order to confirm this indirect evidence.

## 6. BoNT/A Could Change the Paradigm of Treatment in Psychiatry

Although there are established therapies for depression, at least one third of patients fail to respond to any combination of oral antidepressants and psychotherapy [62]. Thus, the development of new treatment approaches for depression is needed.

BoNT/A therapy has several potential advantages as a psychiatry therapeutic. First, a single treatment typically takes only 15 min to administer in an outpatient office setting. No special equipment is required. The ease of delivery and administration is important. Multiple medical specialists have experience in injection, including neurologists, primary care physicians, dermatologists, plastic surgeons, ear nose and throat surgeons, nurse practitioners, and physician assistants. This would greatly facilitate the uptake of BoNT/A upon FDA approval for a psychiatric condition.

Second, it lasts on average about three months, allowing fewer office visits for both patients and physicians and thereby enhancing therapeutic adherence. The long duration of BoNT/A effects allows it to potentially have a role as a depot drug for depression. This would simplify the treatment protocol and reduce the problem of withdrawal symptoms that are sometimes experienced by patients who abruptly stop their oral antidepressant medication. Real-life clinical experience with BoNT/A in depression has shown that depressive symptoms begin to return 2–4 months after injections, in most patients. The duration of action may reflect the dose used versus the strength of the glabellar frown muscles [63]. It has been observed anecdotally that certain individuals with more prominent frown muscles require higher doses for the antidepressant effect to last 3 months [64].

Third, BoNT/A has been used for more than 30 years for a variety of therapeutic and cosmetic indications in millions of patients. This experience has shown that BoNT/A is a safe and well-tolerated treatment for glabellar frown muscles [65]. Side effects usually consist of short-term local irritation, headache, and occasional ptosis.

The optimal dosage of BoNT/A for the treatment of depression has not been fully established. One could argue that some negative emotional activity is necessary for adaptive human behavior. BoNT/A treatments of the frown musculature appear to diminish amygdala activity in normal subjects. This has not been observed to be a difficulty, in terms of behavior, for the millions of nondepressed subjects who have received treatments over many years. In the depressed patient population, BoNT/A is probably normalizing dysfunctional amygdala activity. There are less data on the treatment of males, but they appear to respond as well as females.

The use of BoNT/A for a variety of indications has grown over the past 20 years. In part, it is possible that the enhancement of emotional wellbeing resulting from glabellar injections may well have contributed to the rapid growth and acceptance of this therapeutic modality [66,67].

Currently approved oral antidepressants may have side effects, such as weight gain, fatigue, or sexual dysfunction [68,69], and 44% of patients discontinue their medication because of these side effects [70]. Thus, the burden of side effects appears to be lower with BoNT/A [8,9]. Other approved treatments, such as electric shock therapy, esketamine, and atypical antipsychotics, have more potential for severe side effects. For example, electric shock therapy can cause significant memory loss, and esketamine may cause hallucinations and dissociation that requires on-site monitoring for hours after administration. It is plausible that this may have played a role in reducing the numbers of patients who are treated with such therapies. Thus, for patients, BoNT/A has potential advantages.

Tachyphylaxis has been reported for many drugs, including oral antidepressants. To date this has not been reported for BoNT/A treatment of depression [63]. However, larger-scale and longer-term clinical trials will be necessary to learn more about the role of BoNT/A in depression.

Conceptually, BoNT/A affects emotional processes in the CNS through the muscles of facial expression, by interruption of the emotional proprioceptive feedback loop. The modulation of amygdala activity by BoNT/A also gives this therapy the potential to treat a wide range of psychiatric disorders. Notably, in addition to MDD, bipolar depression, panic disorder, post-traumatic stress disorder, borderline personality disorder, social anxiety disorder, and obsessive–compulsive disorder all demonstrate amygdala dysregulation. Preliminary studies have shown that borderline personality disorder, bipolar depression, and social anxiety disorder can be helped by glabellar BoNT injections [64,71,72,73]. It will, therefore, be of interest to determine a potential role for BoNT/A in psychiatric disorders in general.

## Data Availability

The data presented in this study are available on request from the corresponding author. The data are not publicly available because the primary data is not anonymous data from the outpatient clinic.

## References

[1] Finzi E., Wasserman E. (2006). Treatment of Depression with Botulinum Toxin A: A Case Series. Dermatol. Surg..

[2] Wollmer M.A., de Boer C., Kalak N., Beck J., Gotz T., Schmidt T., Hodzic M., Bayer U., Kollmann T., Kollewe K. (2012). Facing depression with botulinum toxin: A randomized controlled trial. J. Psychiatr. Res..

[3] Finzi E., Rosenthal N.E. (2014). Treatment of depression with onabotulinumtoxinA: A randomized, double-blind, placebo controlled trial. J. Psychiatr. Res..

[4] Magid M., Reichenberg J.S., Poth P.E., Robertson H.T., LaViolette A.K., Kruger T.H.C., Wollmer M.A. (2014). Treatment of major depressive disorder using botulinum toxin A: A 24-week randomized, double-blind, placebo-controlled study. J. Clin. Psychiatry.

[5] Zamanian A., Ghanbari Jolfaei A., Mehran G., Azizian Z. (2017). Efficacy ofIx versus Placebo for Treatment of Patients with Major Depression. Iran. J. Public. Health.

[6] Brin M.F., Durgam S., Lum A., James L., Liu J., Thase M.E., Szegedi A. (2020). OnabotulinumtoxinA for the treatment of major depressive disorder: A phase 2 randomized, double-blind, placebo-controlled trial in adult females. Int. Clin. Psychopharmacol..

[7] Dong H., Fan S., Luo Y., Peng B. (2018). Botulinum toxin relieves anxiety and depression in patients with hemifacial spasm and blepharospasm. Neuropsychiatr. Dis. Treat..

[8] Zhang H., Zhang H., Wei Y., Lian Y., Chen Y., Zheng Y. (2017). Treatment of chronic daily headache with comorbid anxiety and depression using botulinum toxin A: A prospective pilot study. Int. J. Neurosci..

[9] Zhu C., Wang K., Yu T., Liu H. (2021). Effects of botulinum toxin type a on mood and cognitive function in patients with Parkinson's disease and depression. Am. J. Transl. Res..

[10] Li Y., Zhu T., Shen T., Wu W., Cao J., Sun J., Liu J., Zhou X., Jiang C., Tang Z. (2022). Botulinum toxin A (BoNT/A) for the treatment of depression: A randomized, double-blind, placebo, controlled trial in China. J. Affect. Disord..

[11] Ceolato C., Charles E., Clément J.P., Ranoux D. (2018). Botulinum toxin in the treatment of resistant depressive disorder: Comparison of 2 facial injection sites. Toxicon.

[12] Darwin C. (1872). The Expression of the Emotions in Man and Animals.

[13] James W. (1890). The Principles of Psychology.

[14] Ekman P., Sorenson E.R., Friesen W.V. (1969). Pan-cultural elements in facial displays of emotion. Science.

[15] Ekman P., Levenson R.W., Friesen W.V. (1983). Autonomic nervous system activity distinguishes among emotions. Science.

[16] Larsen R.J., Kasimatis M., Frey K. (1992). Facilitating the Furrowed Brow: An Unobtrusive Test of the Facial Feedback Hypothesis Applied to Unpleasant Affect. Cogn. Emot..

[17] Zajonc R.B., Murphy S.T., Inglehart M. (1989). Feeling and facial efference: Implications of the vascular theory of emotion. Psychol. Rev..

[18] Adelmann P.K., Zajonc R.B. (1989). Facial efference and the experience of emotion. Ann. Rev. Psychol..

[19] Heller A.S., Greischar L.L., Honor A., Anderle M.J., Davidson R.J. (2011). Simultaneous acquisition of corrugator electromyography and functional magnetic resonance imaging: A new method for objectively measuring affect and neural activity concurrently. NeuroImage.

[20] Heller A.S., Lapate R.C., Mayer K.E., Davidson R.J. (2014). The face of negative affect: Trial-by-trial corrugator responses to negative pictures are positively associated with amygdala and negatively associated with ventromedial prefrontal cortex activity. J. Cogn. Neurosci..

[21] Schwartz G.E., Fair P.L., Salt P., Mandel M.R., Klerman G.L. (1976). Facial muscle patterning to affective imagery in depressed and nondepressed subjects. Science.

[22] Whatmore G.B., Ellis R.M. (1959). Some neurophysiologic aspects of depressed states: An electromyographic study. AMA Arch. General. Psychiatry.

[23] Whatmore G.B., Ellis R.M. (1962). Further Neurophysiologic Aspects of Depressed States: An Electromyographic Study. Arch. General. Psychiatry.

[24] Carney R.M., Hong B.A., O’Connell M.F., Amado H. (1981). Facial electromyography as a predictor of treatment outcome in depression. Br. J. Psychiatry.

[25] Schwartz G.E., Fair P.L., Mandel M.R., Salt P., Mieske M., Klerman G.L. (1978). Facial electromyography in the assessment of improvement in depression. Psychosom. Med..

[26] Greden J.F., Price H.L., Genero N., Feinberg M., Levine S. (1984). Facial EMG activity levels predict treatment outcome in depression. Psychiatry Res..

[27] Finzi E. (2013). The Face of Emotion: How Botox Affects Moods and Relationships.

[28] Finzi E. (2013). Antidepressant effects of botulinum toxin A: Scientific rationale. J. Psychiatry Neurosci..

[29] Finzi E., Rosenthal N.E. (2016). Emotional proprioception: Treatment of depression with afferent facial feedback. J. Psychiatr. Res..

[30] Finzi E. (2018). Update: Botulinum Toxin for Depression: More Than Skin Deep. Dermatol. Surg..

[31] Matsuo K., Ban R., Hama Y., Yuzuriha S. (2015). Eyelid opening with tri- geminal proprioceptive activation regulates a brainstem arousal mechanism. PLoS ONE.

[32] Lanteaume L., Khalfa S., Régis J., Marquis P., Chauvel P., Bartolomei F. (2007). Emotion induction after direct intracerebral stimulations of human amygdala. Cereb. Cortex.

[33] Loureiro J.R.A., Leaver A., Vasavada M., Sahib A.K., Kubicki A., Joshi S., Woods R.P., Wade B., Congdon E., Espinoza R. (2020). Modulation of amygdala reactivity following rapidly acting interventions for major depression. Hum. Brain Mapp..

[34] Ressler K.J., Berretta S., Bolshakov V.Y., Rosso I.M., Meloni E.G., Rauch S.L., Carlezon W.A. (2022). Post-traumatic stress disorder: Clinical and translational neuroscience from cells to circuits. Nat. Rev. Neurol..

[35] Minkova L., Sladky R., Kranz G.S., Woletz M., Geissberger N., Kraus C., Lanzenberger R., Windischberger C. (2017). Task-dependent modulation of amygdala connectivity in social anxiety disorder. Psychiatry Res. Neuroimaging.

[36] Bigot M., Alonso M., Houenou J., Sarrazin S., Dargél A.A., Lledo P.M., Henry C. (2020). An emotional-response model of bipolar disorders integrating recent findings on amygdala circuits. Neurosci. Biobehav. Rev..

[37] Quagliato L.A., Carta M.G., Nardi A.E. (2022). Panic Disorder Seeks More Specific Drugs for Treatment: Might the Amygdala Be the Best Target?. J. Clin. Psychopharmacol..

[38] Geurts D.E.M., Van den Heuvel T.J., Huys Q.J.M., Verkes R.J., Cools R. (2022). Amygdala response predicts clinical symptom reduction in patients with borderline personality disorder: A pilot fMRI study. Front. Behav. Neurosci..

[39] Cao L., Li H., Hu X., Liu J., Gao Y., Liang K., Zhang L., Hu X., Bu X., Lu L. (2022). Distinct alterations of amygdala subregional functional connectivity in early- and late-onset obsessive-compulsive disorder. J. Affect. Disord..

[40] Ruhé H.G., Booij J., Veltman D.J., Michel M.C., Schene A.H. (2012). Successful pharmacologic treatment of major depressive disorder attenuates amygdala activation to negative facial expressions: A functional magnetic resonance imaging study. J. Clin. Psychiatry.

[41] Hennenlotter A., Dresel C., Castrop F., Ceballos-Baumann A.O., Wohlschläger A.M., Haslinger B. (2009). The link between facial feedback and neural activity within central circuitries of emotion-new insights from botulinum toxin-induced denervation of frown muscles. Cereb. Cortex.

[42] Kim M.J., Neta M., Davis F.C., Ruberry E.J., Dinescu D., Heatherton T.F., Stotland M.A., Whalen P.J. (2014). Botulinum toxin-induced facial muscle paralysis affects amygdala responses to the perception of emotional expressions: Preliminary findings from an A-B-A design. Biol. Mood Anxiety Disord..

[43] Kruger T.H.C., Schulze J., Bechinie A., Neumann I., Jung S., Sperling C., Engel J., Müller A., Kneer J., Kahl K.G. (2022). Neuronal effects of glabellar botulinum toxin injections using a valenced inhibition task in borderline personality disorder. Sci. Rep..

[44] James W. (1884). What is an Emotion?. Mind.

[45] Cannon W.B. (1927). The James-Lange theory of emotions: A critical examination and an alternative theory. Am. J. Psychol..

[46] Dror O.E. (2013). The Cannon–Bard Thalamic Theory of Emotions: A Brief Genealogy and Reappraisal. Emot. Rev..

[47] Laird J.D., Lacasse K. (2014). Bodily influences on emotional feelings: Accumulating evidence and extensions of William James’s theory of emotion. Emot. Rev..

[48] Niedenthal P.M. (2007). Embodying emotion. Science.

[49] Soussignan R. (2022). Duchenne smile, emotional experience, and autonomic reactivity: A test of the facial feedback hypothesis. Emotion.

[50] Dolensek N., Gehrlach D.A., Klein A.S., Gogolla N. (2020). Facial expressions of emotion states and their neuronal correlates in mice. Science.

[51] Antipova V., Holzmann C., Hawlitschka A., Witt M., Wree A. (2021). Antidepressant-Like Properties of Intrastriatal Botulinum Neurotoxin-A Injection in a Unilateral 6-OHDA Rat Model of Parkinson's Disease. Toxins.

[52] Li Y., Liu J., Liu X., Su C.J., Zhang Q.L., Wang Z.H., Cao L.F., Guo X.Y., Huang Y., Luo W. (2019). Antidepressant-Like Action of Single Facial Injection of Botulinum Neurotoxin A is Associated with Augmented 5-HT Levels and BDNF/ERK/CREB Pathways in Mouse Brain. Neurosci. Bull..

[53] Chen W.J., Niu J.Q., Chen Y.T., Deng W.J., Xu Y.Y., Liu J., Luo W.F., Liu T. (2021). Unilateral facial injection of Botulinum neurotoxin A attenuates bilateral trigeminal neuropathic pain and anxiety-like behaviors through inhibition of TLR2-mediated neuroinflammation in mice. J. Headache Pain..

[54] Arnone D., Galadari H., Rodgers C.J., Ostlundh L., Aziz K.A., Stip E., Young A.H. (2021). Efficacy of onabotulinumtoxinA in the treatment of unipolar major depression: Systematic review, meta-analysis and meta-regression analyses of double-blind randomised controlled trials. J. Psychopharmacol..

[55] Schulze J., Neumann I., Magid M., Finzi E., Sinke C., Wollmer M.A., Kruger T.H.C. (2021). Botulinum toxin for the management of depression: An updated review of the evidence and meta-analysis. J. Psychiatr. Res..

[56] Crowley J.S., Silverstein M.L., Reghunathan M., Gosman A.A. (2022). Glabellar Botulinum Toxin Injection Improves Depression Scores: A Systematic Review and Meta-Analysis. Plast. Reconstr. Surg..

[57] Wollmer M.A., Magid M., Kruger T.H.C., Finzi E. (2021). The Use of Botulinum Toxin for Treatment of Depression. Handb. Exp. Pharmacol..

[58] Affatato O., Moulin T.C., Pisanu C., Babasieva V.S., Russo M., Aydinlar E.I., Torelli P., Chubarev V.N., Tarasov V.V., Schiöth H.B. (2021). High efficacy of onabotulinumtoxinA treatment in patients with comorbid migraine and depression: A meta-analysis. J. Transl. Med..

[59] Cohen I.V., Makunts T., Atayee R., Abagyan R. (2017). Population scale data reveals the antidepressant effects of ketamine and other therapeutics approved for non-psychiatric indications. Sci. Rep..

[60] Makunts T., Wollmer M.A., Abagyan R. (2020). Postmarketing safety surveillance data reveals antidepressant effects of botulinum toxin across various indications and injection sites. Sci. Rep..

[61] Wollmer M.A., Makunts T., Krüger T.H.C., Abagyan R. (2021). Postmarketing safety surveillance data reveals protective effects of botulinum toxin injections against incident anxiety. Sci. Rep..

[62] Rush A.J., Trivedi M.H., Wisniewski S.R., Nierenberg A.A., Stewart J.W., Warden D., Niederehe G., Thase M.E., Lavori P.W., Lebowitz B.D. (2006). Acute and longer-term outcomes in depressed outpatients requiring one or several treatment steps: A STAR*D report. Am. J. Psychiatry.

[63] Lehnert F., Neumann I., Kruger T.H.C., Magid M., Wollmer M.A. (2023). Botulinum toxin therapy for psychiatric disorders in clinical practice: A retrospective case study. Toxins.

[64] Finzi E., Kels L., Axelowitz J., Shaver B., Eberlein C., Krueger T.H.C., Wollmer M.A. (2018). Botulinum toxin therapy of bipolar depression: A case series. J. Psychiatr. Res..

[65] Brin M.F., Boodhoo T.I., Pogoda J.M., James L.M., Demos G., Terashima Y., Gu J., Eadie N., Bowen B.L. (2009). Safety and tolerability of onabotulinumtoxinA in the treatment of facial lines: A meta-analysis of individual patient data from global clinical registration studies in 1678 participants. J. Am. Acad. Dermatol..

[66] Sommer B., Zschocke I., Bergfeld D., Sattler G., Augustin M. (2003). Satisfaction of patients after treatment with botulinum toxin for dynamic facial lines. Dermatol. Surg..

[67] Sykianakis D., Stratigos A., Chatziioannou A., Christodoulou C. (2022). Botulinum toxin type A treatment is associated with improved social and psychological behavior: A retrospective study. J. Cosmet. Dermatol..

[68] Khawam E.A., Laurencic G., Malone D.A. (2006). Side effects of antidepressants: An overview. Clevel. Clin. J. Med..

[69] Kim M.J., Neta M., Davis F.C., Ruberry E.J., Dinescu D., Heatherton T.F., Stotland M.A., Whalen P.J., Rothmore J. (2020). Antidepressant-induced sexual dysfunction. Med. J. Aust..

[70] Lin E.H., Von Korff M., Katon W., Bush T., Simon G.E., Walker E., Robinson P. (1995). The role of the primary care physician in patients’ adherence to antidepressant therapy. Med. Care.

[71] Wollmer M.A., Neumann I., Jung S., Bechinie A., Herrmann J., Muller A., Wohlmuth P., Fournier-Kaiser L., Sperling C., Peters L. (2022). Clinical effects of glabellar botulinum toxin injections on borderline personality disorder: A randomized controlled trial. J. Psychopharmacol..

[72] Kruger T.H.C., Magid M., Wollmer M.A. (2016). Can Botulinum Toxin Help Patients with Borderline Personality Disorder?. Am. J. Psychiatry.

[73] Finzi E., Rosenthal N.E. (2019). Botulinum Toxin Therapy of Social Anxiety Disorder: A Case Series. J. Clin. Psychopharmacol..

